# Children’s Expressions of Worry During the COVID-19 Pandemic in Sweden

**DOI:** 10.1093/jpepsy/jsab060

**Published:** 2021-08-17

**Authors:** Anna Sarkadi, Lisa Sahlin Torp, Anna Pérez-Aronsson, Georgina Warner

**Affiliations:** Child Health and Parenting (CHAP), Department of Public Health and Caring Sciences, Uppsala University

**Keywords:** adolescents, COVID-19, qualitative methods, school-age children

## Abstract

**Objective:**

Sweden is an international exception in its public health response to the COVID-19 pandemic, with a higher number of deaths, albeit not pediatric, compared with other Nordic countries. The objective of this study was to investigate what worries children and adolescents living in Sweden expressed in relation to the pandemic.

**Methods:**

Using an anonymous web-survey, 1,047 children (4–12 years; *N* = 717) and adolescents (13–18 years; *N* = 330) responded to five background and four open-ended questions, one of which was: *Is there anything that you are worried about when it comes to ‘Corona’?* The responses were coded using manifest content analysis. Interrater reliability was .95, assessed on the code level.

**Results:**

Worry was common (77%); mostly (60%) related to disease or death of elderly relatives, parents, the child him/herself or general worry for the elderly/risk groups. Existential worry (15%) comprised worries about the future, including economy and worries about the world perishing or the contagion becoming uncontrollable. A developmental trajectory was evident in the nature of responses. Adolescents’ worries about the future included missing out on their youth and employment. They also worried about society (6%), for example, the future of democracy and the world economy. There was no indication of socioeconomic status or geographic area (urban vs. rural) affecting the presence of worrisome thoughts.

**Conclusions:**

Worry about “Corona” was common. Universal preventative mental health intervention is warranted and could be conducted in the school setting. Intervention could be tailored by age, covering discussion on financial aspects with adolescents.

## Introduction

COVID-19 is a disease caused by a novel coronavirus calledSARS-CoV-2. The World Health Organization (WHO) first learned of this new virus in December 2019, following a report of a cluster of cases of “viral pneumonia” in Wuhan, People’s Republic of China (World Health Organization, 2020). As of January 2021, there had been over 90 million confirmed cases and over 2 million deaths worldwide (World Health Organization, 2020). In Sweden, there were over 500 thousand confirmed cases and over 10,000 deaths (The Swedish Public Health Agency, 2020a).

Sweden has been an international exception in its public health response to the pandemic with no lockdown at any stage. At the time of this study (April–May 2020), only prohibition of gatherings for more than 50 people (Regeringen, 2020) voluntary restrictions on travels and well-endorsed recommendations on social distancing and working from home, when possible, were in place (Socialstyrelsen, 2020). The Swedish Public Health Agency (2021) explicitly stated that the reason to keep preschools and primary schools open was their protective role for child public health. This left many children’s everyday lives less affected by restrictions. For high school-aged youth, 16–18 years, the situation was different with exclusively online education from mid-March to June 2020 (The Swedish Public Health Agency, 2020b). The Swedish strategy has been widely discussed given a higher number of deaths, albeit not pediatric, compared with other Nordic countries (Ludvigsson, 2020). Since early April, there have been daily reports on morbidity and mortality, and repetition of the recommendations on daily press conferences held by the National Public Health Agency, stressing the need to protect the elderly (Claeson & Hanson, 2021; Ludvigsson, 2020). The high number of reported deaths in aged care facilities and constant discussions about failure to protect the elderly in the media has become the backdrop to everyday life. Thus, on the one hand, Swedish children’s school routines were relatively undisrupted, unlike in many other countries, including the Nordic neighbors. On the other hand, Sweden’s death rates clearly stood out compared with other Nordic countries. What would the sum of these societal circumstances be on children’s experiences of worry during the pandemic in Sweden?

There have been a number of perspective pieces published on the potential negative effects of COVID-19 on child mental health since the onset of the pandemic (Coller & Webber, 2020; Golberstein et al., 2020; Lee, 2020; Liu et al., 2020a). These concerns have been confirmed by research, with the negative effects of the pandemic on children’s well-being highlighted by various surveys of parents and children. A survey of parents in China reported a number of observed negative health and well-being behaviors among children (Jiao et al., 2020). Parent-report surveys conducted in the United States have shown adverse effects of COVID-19 on child well-being, comprising apparent negative impact on both behavioral and physical health, including perceived worry (Gassman-Pines et al., 2020; Patrick et al., 2020). Child and adolescent self-report surveys have also been conducted in China, which demonstrated raised levels of anxiety and depressive symptoms (Duan et al., 2020; Liu et al., 2020b; Zhou et al., 2020), particularly among girls, children residing in urban regions, and children with an emotion-focused coping style (Duan et al., 2020). Yet, the well-being of Swedish children and adolescents, who have experienced relatively unusual COVID-19 societal circumstances given the pandemic management decisions adopted in Sweden, remains under-researched.

The aim of this study was to investigate what kind of worries, if any, children and adolescents living in Sweden expressed in relation to the pandemic. This study was particularly unique in that it focused on the cognitive construct of worry rather than clinical measures of anxiety and/or depression. There is a growing recognition that we cannot provide effective support services by waiting for children to develop clinical symptoms (Hess et al., 2013). Instead, services that recognize and deal with early signs of developing problems should be integrated in schools and other community settings (Hess et al., 2013). In the case of the COVID-19 pandemic, the full impact on children’s mental health may not become clear for some time, yet early intervention efforts can begin. Although worry is a common phenomenon among children (Muris et al., 1998; Orton, 1982), the Diagnostic and Statistical Manual of Mental Disorders posits worry as a key feature of Generalized Anxiety Disorder (American Psychiatric Association, 2013). Therefore, it is a logical early intervention target and understanding more about the nature of children’s COVID-19-related worries can inform intervention efforts.

The study utilized an anonymous survey with open-ended questions rather than standardized questionnaires. This is another point of departure from extant literature. To date, COVID-19 surveys with youth have utilized quantitative methods. Duan et al. (2020) utilized regression analysis to consider which child characteristics were associated with increased levels of anxiety among children in China and, similarly, Gassman-Pines et al. (2020) considered associations between COVID-19 hardships and perceived child well-being. This form of analysis can give some insight to possible mechanisms behind children’s worrisome thoughts, but it is somewhat limited as only predetermined factors can be included in the model. Qualitative design, on the other hand, gives children space to provide detail about their worries in their own words and enables the nuanced perspectives of children across various ages to be captured. This study coupled qualitative analysis with simple quantitative analysis comparing children’s responses according to their demographics. As the survey was explorative, there were no specific hypotheses. However, it was anticipated that at least some children in Sweden would have experienced worrisome thoughts about COVID-19 and, given the medical profile and high mortality rate of COVID-19, worry related to disease or death was expected.

## Materials and Methods

This study was an anonymous cross-sectional internet-based survey asking children and adolescents in Sweden between the ages of 4 and 18 years open-ended questions about their thoughts and reactions to COVID-19, including any worries they have. To encourage wide participation, the study was advertised through various media outlets and the survey was available in four languages with pictorial support. Parents were encouraged to assist their children aged 4–12 years in the completion of the survey.

### Participants

Aside from age and country of residence, ability to complete the survey was the only eligibility requirement. In total, 1,106 children (4–12 years; *N* = 748) and adolescents (13–18 years; *N* = 358) took part in the study, of which 1,047 children (4–12 years; *N* = 717) and adolescents (13–18 years; *N* = 330) provided responses about worrisome thoughts. The demographics of those who responded to the question about worrisome thoughts are shown in Table I. Respondents were 4-18 years old (*M* = 10.6; SD = 3.9). Responses were received from all 21 counties in Sweden; however, some counties (Gotland County and Uppsala County) were overrepresented and others (Stockholm County, Västra Götaland County and Skåne County) underrepresented based on population statistics. In terms of rural or urban living, 460 (44%) reported living in a larger city, 305 (29%) in a smaller city, and 280 (27%) in a smaller town or the countryside. Most children (*N* = 712; 68%) reported living in a house, 157 (15%) reported living in an owned apartment and 177 (17%) in a rented apartment.

**Table I.** Demographics of Respondents to the Question About Worrisome Thoughts

Characteristic	*N*	%
Age (years)		
4	58	6
5	73	7
6	65	6
7	68	7
8	72	7
9	75	7
10	93	9
11	123	12
12	90	9
13	68	7
14	68	7
15	64	6
16	48	5
17	49	5
18	33	3
County		
Blekinge	26	3
Dalarna	5	1
Gävleborg	4	0
Gotland	187	18
Halland	53	5
Jämtland	23	2
Jönköping	42	4
Kalmar	12	1
Kronoberg	52	5
Norrbotten	6	1
Örebro	20	2
Östergötland	18	2
Skåne	48	5
Södermanland	11	1
Stockholm	191	18
Uppsala	175	17
Värmland	11	1
Västerbotten	19	2
Västernorrland	7	1
Västmanland	27	3
Västra Götaland	108	10
Housing type		
House	712	68
Owned apartment	157	15
Rented apartment	177	17
Rural or urban living		
Larger city	460	44
Smaller city	305	29
Smaller town or countryside	280	27

### Recruitment

The survey was distributed nationally and locally, through newspapers (mostly local papers, e.g., Katrineholms-Kuriren, Värmlands Folkblad, Ystads Allehanda, Sydnärkenytt, Dala-Demokraten, following the local press releases that were sent to all the 21 counties), radio (e.g., Sveriges Radio), television broadcasts (e.g., SVT Morgonstudion), and social media (e.g., Facebook). The press releases for the study were managed by a public relations (PR) company recruited by the study funder, in collaboration with the research team and the university press office. This coverage was primarily in Swedish; however, social media posts were made in other languages (Arabic, English, and Somali) by native research group speakers. An example of the messaging was: “Many children and young people are anxious and have thoughts about corona, but their voices are rarely heard. Länsförsäkringar’s Research Fund therefore funds a new research study at Uppsala University to investigate children’s and young people’s views of corona.” The PR company estimated potential reach of 2.5 million people for the campaign, which is about a fourth of the Swedish population. Almost all Swedish counties had some form of local coverage. Children and youth could donate ∼5 USD at the end of the survey to one of four NGOs working for children’s health and/or rights, as a form of reimbursement, financed by the study sponsor. Advice to carers on how to talk to children about the pandemic was also distributed in connection to the press release about the study and on the website for the study.

### Consent/Assent Procedures

The Swedish legislation on ethical review for research involving human subjects (SFS 2003:460) stipulates that it applies to “research involving the processing of personal data referred to in article 9 in the EU data protection regulation (sensitive personal data) or personal data on offenses involving crime, criminal convictions, coercive measures or administrative detentions.” The survey of this study was anonymous and no personal details about the participants were sought or stored. The brief background questions included in the survey were carefully designed to be broad and not leading to a risk of identifying individual participants; only age, type of housing, and region were asked for. Thus, the survey could be conducted without ethical review but with consideration to ethical principles. The project home page hosted ‘buttons' that linked each visitor type (4–12 years old; 13–18 years old; parent; educational personnel) to a dedicated webpage. Parents were asked to fill in the survey together with their children up to 12 years of age. This was made explicit on both the parent webpage and the child webpage. Adolescents were encouraged to fill in the questionnaire on their own, but for 13 and 14 year olds a prompt was sent to discuss concerns or worries with an adult, should they have any, after completing the survey. On the first page of the survey the participant was presented with an information statement, which covered: who was conducting the research; the purpose of the study; that they could select from a list of organizations that help children and young people at the end of the survey to which the project funder would make a donation; and that their answers were anonymous.

### Measures

The survey contained nine questions for 4–12 year olds and 16 questions for 13–18 year olds. The survey was made available in four different languages (Swedish, Arabic, English, and Somali) as well as in a version with pictorial support for users of augmentative and alternative communication. The survey content was first drafted in Swedish; then, translated by members of the research group native in the other languages. The translations were available in portable document format (PDF) on the study webpage as well as survey links. The various language survey links were not utilized; only Swedish survey data were included. Background questions included age, county, rural or urban living, housing type (rental flat, owned flat, or house), and acquaintance of someone with COVID-19. Housing type was used as a proxy for socioeconomic status given that high quality official statistics are available on housing type for children of different ages and the link between housing type and income in different Swedish regions. The children had the option to select “not sure” in response to the question about knowing someone with COVID-19. This was due to the fact they may not have communicated with others about COVID-19 and thus could be unaware of an acquaintance’s diagnosis. We chose not to ask participants for their sex. The reasoning for this was threefold: to limit the amount of potentially identifiable or sensitive data to comply with ethical requirements for anonymous surveys; sex- or gender-based analysis was not included in the study plan; and to minimize the question burden of the survey to promote the response rate. We used four open-ended questions, which were piloted on 50 children 3.5–18 years of age and youth prior to launching the survey. In this study, we report answers to one of the four open-ended questions: *Is there anything that you are worried about when it comes to “Corona”?* The other questions posed were: *What do you think about “Corona”? What is the best thing about “Corona” for you? What is the worst thing about “Corona” for you?* All of the questions were presented as text response questions, with large text fields to prompt detailed responses.

### Data Collection

An anonymous web-survey was posted on a designated study website hosted by Uppsala University. Different versions of the survey were hosted on the 4–12 and 13–18 years old webpages. Translated versions of the surveys were made available via separate links for both age groups. Additionally, PDF versions of the survey questions in all available languages were available on the webpages. There were links provided to websites for information about COVID-19 as well as access to helplines and children’s rights organizations on the project website. Parents were also provided with links to guidance on how to talk to children about the pandemic. Data were stored in the survey interface Qualtrics, where anonymous entry was chosen and thus no Internet Protocol addresses or other identifying information were registered.

### Data Analytic Approach

The responses were coded using manifest content analysis (Graneheim & Lundman, 2004). The survey responses were read through several times to obtain a sense of the whole. Then the responses to the question *“Is there anything that you are worried about when it comes to ‘Corona’?”* were extracted to a dedicated excel file. The responses were divided into meaning units that were condensed. The condensed meaning units were abstracted and labeled with a code. The whole context was considered when condensing and labeling meaning units with codes. Interrater reliability was .95, assessed on the code level. The various codes were compared based on differences and similarities and sorted into categories, which constitute the manifest content. The tentative categories were discussed by two researchers (A.S. and L.S.T.) and revised. A process of reflection and discussion resulted in agreement about how to sort the codes. Finally, the underlying meaning, that is, the latent content, of the categories was formulated into themes.

Descriptive statistics that is, absolute numbers and percentages, were used to quantitatively summarize how many children reported worries in each of the themes and whether or not they knew anyone who has become ill with COVID-19. Associations between presence of worry and (a) housing type (proxy for socioeconomic status), (b) rural or urban living (smaller city; larger city; smaller town; or countryside), and (c) knowing someone with COVID-19 were explored using Chi-square tests of independence. Cramer’s *V* was calculated to indicate effect size. A logistic regression was performed to ascertain the effect of age on the presence of worry, and the odds ratio calculated.

## Results

**Figure 1.**
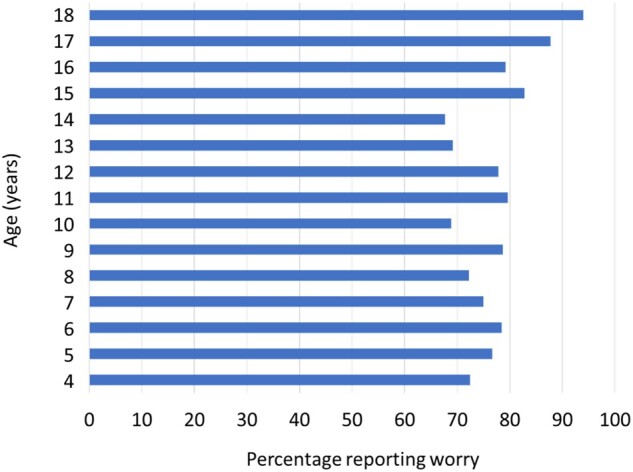
Proportion of children reporting worrisome thoughts by age.

Worry was common (77%) and consistently reported across the age range of the study (Figure 1). Age was not significantly associated with the likelihood of worrisome thoughts, *B* = 0.034, SE = 0.019, Wald = 3.241, *p* = .072, odds ratio = 1.035 (95% CI: 0.997–1.074). There was no relationship between housing type, taken as a proxy of socioeconomic status, and the presence of worry, *X*^2^ (2, *N* = 1,047) = 4.232, *p* = .120, Cramer’s *V* = .064. Nor was there an association with rural or urban living (smaller city; larger city; smaller town; or countryside), *X*^2^ (3, *N* = 1,047) = 5.451, *p* = .142, Cramer’s *V* = .072. When asked if the child or adolescent knew anyone who has become ill with the corona virus, 299 (28%) responded “yes,” 444 (43%) responded “no” and 304 (29%) were not sure. There was no significant relationship between knowing someone with COVID-19 and the presence of worry, *X*^2^ (3, *N* = 743) = 3.458, *p* = 0.063, Cramer’s *V* = .068.

The results from the content analysis, including themes, proportion of respondents within each theme, categories, and codes are shown in Table II.

**Table II.** Results From Qualitative Content Analysis of Children and Adolescents’ Answers to the Question: Is There Anything About Corona That You’re Worried About?

Themes	% children (4–12 years)	% adolescents (13–18 years)	Categories	Codes
No worry	24	21	*No worry*	No worry
Worry about disease or death	63	56	*Worry about getting sick/dying*	Worry getting sick
				Worry dying
			*Worry about loved ones getting sick/dying*	Worry about disease/death grandparents
				Worry about disease/death parents/siblings
			*Worry about disease/death elderly/risk group*	Worry about disease/death in elderly/risk group
				Worry about disease/death in others
Existential worries	13	17	*Worry about future*	Long term impact day to day life^a^
				Economy^a^
				Never ends/returns to normal
				Hard to get a job^b^
			*Worry about unstoppable infection*	Whole world gets infected
				No cure is found
				Everyone dies^a^
				Corona as a creature^a^
				Infecting others
				Youths/children get infected too^b^
			*Missing out* ^b^	Missing out on youth/graduation^b^
				Missing out on youth^b^
				“Quarantine” for a long time^b^
Worry for society^b^	0	6	*General worry for society* ^b^	Too much strain for the healthcare system
				Negative impact on trust in politicians/democracy^b^
				Global economy^b^

a Only present in child (4–12 years old) analysis.

b Only present in adolescent (13–18 years old) analysis.

### Worry About Disease or Death

Worrisome thoughts about disease or death were very common among the respondents, both children and adolescents. These expressions of worry related to various people. Some respondents expressed worry about getting sick, or dying, themselves.

I’m worried that I’m going to die. (7 year old)

Whereas, some described being worried about others getting sick or dying. A number of children conveyed worry about loved ones getting sick. This mostly related to grandparents, but also extended to parents and siblings.

I know I won't be so dangerously infected, but I hope that my older relatives will not be infected because they are of course at risk so they can actually die. (9 year old)

I'm partly worried that my loved ones will get sick. My older relatives and parents mean an incredible amount to me and I am of course worried that they will fall ill, or even lose their life to Corona. (17 year old)

Some respondents expressed concern about others in society, which largely related to the known risk group of elderly people.

That very, very, very many old ones die. (4 year old)

I am most worried about the elderly who are having a hard time. (14 year old)

### Existential Worries

Existential worries were interpreted as a theme for both children and adolescents. However, the nature of worrisome thoughts within this theme varied between the age groups. Both age groups expressed worries about the future. The younger respondents communicated worries about the long-term impact on day-to-day life. They also made comments relating to economy, particularly family-level economy such as parental employment.

That we children are left alone when old people die. But mum says we will not be. (5 years old)

I worry my parents are going to lose their jobs. (14 years old)

The adolescent respondents spoke specifically about their own employment prospects, expressing that they worried it would be difficult for them to find a job in the future due to the pandemic.

I am worried about my future as I do not know if I will be able to get a job after graduation and so will not be able to move away from home. (18 years old)

Both age groups conveyed worry about the infection being unstoppable. Respondents of all ages expressed concern that the whole world would get infected, and both age groups shared the concern that no cure would be found. It was also common across the ages to worry about infecting others. Among the adolescent respondents, there was a common worry that children and young people would become a risk group. Children conveyed worry about everyone dying, and some of the youngest children described corona as a creature.

I worry that it will wipe out the whole world. (14 years old)

If corona takes over the whole Earth and gets even worse. So even the animals and children and babies can get corona. Then the astronauts do not want to return to Earth because there is corona. That's what I'm worried about. (7 years old)

There is a super-terrifying Corona with no body that eats people. (4 years old)

Worry about “missing out” emerged as a category among the adolescent responses. They spoke of missing out on their youth, with several specifically referring to missing their graduation. They also referred to being in quarantine for a long time and the impact this would have on them missing social activities.

…another thing I worry about is that the restrictions will be long-lasting. Right now I feel like I'm “missing” my youth and I don't want to have to do it for years. (17 years old)

### Worry for Society

A final category, exclusively seen in the adolescent responses, was general worry for society. Several expressed worrisome thoughts about the strain corona was having on the Swedish healthcare system. Some also spoke of the potential impact on democracy, specifically depleting trust in politicians due to pandemic management choices. Finally, some conveyed concern about the impact the pandemic was having on the global economy.

I am concerned about the overload that may affect the health care system if people do not take responsibility and limit their contacts and how the health care system and mainly its staff are affected by long-term stress. (17 years old)

I am concerned about how all the countries of the world are affected by the economy, as people are very limited in terms of jobs. (15 years old)

I am worried that we will never be able to get back from this, that trust in politicians and democracy will be permanently damaged. (17 years old)

## Discussion

This study investigated the worrisome thoughts of children and adolescents living in Sweden expressed in relation to the ongoing COVID-19 pandemic. The findings indicate that worrisome thoughts about COVID-19 are common and mostly related to disease or death. Existential worry was also evident and comprised worries about the future, including about the economy, the world perishing or contagion becoming uncontrollable. A developmental trajectory was evident in the nature of responses. Adolescents’ worries about the future included missing out on their youth and employment. They also worried about society, such as the future of democracy and the world economy. These findings offer a unique contribution to the extant literature on COVID-19, as there has been a paucity of research into the well-being of Swedish children and adolescents, who have experienced relatively unusual COVID-19 societal circumstances given the pandemic management decisions adopted in Sweden.

It is not surprising that high levels of worry were reported by the children and adolescents. Worry is commonly reported among children internationally; community samples of children surveyed prior to COVID-19 show that around 70% report experiencing worry (Muris et al., 1998; Orton, 1982). School performance, health, social contacts, and personal harm are among the most common worries reported by children (Muris et al., 1998; Silverman et al., 1995). As COVID-19 has had a direct impact on people’s health and has indirectly impacted school attendance (only for adolescents in Sweden) and social contact, it is logical that children have experienced worrisome thoughts about COVID-19 given their typical profile of worries. General worry among children during the COVID-19 pandemic has also been reported by parents in China (Jiao et al., 2020).

Given the medical profile and high mortality rate of COVID-19, worry related to disease or death was anticipated. It has been heavily reported in the media that older people are a high risk group for COVID-19 and so the children describing worries about the elderly, including their own grandparents, getting sick is to be expected. Again, this aligns with parent reports from China, which highlighted a fear for the health of relatives among children (Jiao et al., 2020). Literature on bereavement indicates that the death of a grandparent can lead to increased death anxiety in children (Ens & Bond, 2005). Some of the children who completed the survey may have lost a grandparent to COVID-19. Although, it may also be the case that widespread news about the death of elderly people, unrelated to the children, has initiated worrisome thoughts about death and feelings of death anxiety. Previous research has indicated that media viewing of tragic events is sufficient to produce trauma symptoms in vulnerable populations such as children (Otto et al., 2007) and exposure to disaster media cues elevates children’s state anxiety (Ortiz et al., 2011). It could therefore be the case that media coverage of COVID-19, which due to the globalized nature of media platforms can draw on pandemic experiences and impacts beyond Sweden, is playing some role in the development of COVID-19 related worrisome thoughts among the children and adolescents in Sweden. Given the lack of relationship between knowing someone with COVID-19 and the presence of worry, it seems feasible the media is playing an instrumental role in the development of worrisome thoughts.

Existential worry can be conceptualized as a reaction to threats to important values or goals that one embraces (May, 1979). The cognitive response of worrying can be perceived by the child as a signal that something vital is being threatened, which has made them reflect upon life and its possibilities (Cooper, 1999). It is interesting that for some children and adolescents this reflection extended beyond health outcomes into other societal worries, such as the impact of COVID-19 on the economy. When considering the context in which the study was conducted, it is notable that economics, including the Swedish tax-funded welfare system, is covered in the school curriculum and so it is likely there is a good level of awareness regarding how nations make choices about how to allocate resources among children in Sweden. There has also been an increase in unemployment rates in Sweden during the pandemic, which children are likely to be aware of due to media coverage.

A developmental trajectory of worry was evident in the nature of responses across the age range of this study (4–18 years). The youngest children utilized story-like narrative to describe their feelings of worry, whereas older children gave more complex responses. These findings replicate those seen in longitudinal research investigating the development of worrisome thoughts throughout childhood (Caes et al., 2016). Caes et al. (2016) describe how, based on mother’s reports (*N* = 2,227), the complexity and elaboration of worrisome thoughts increases from around 8 years. However, in the longitudinal study worry appears to peak around the age of 10 years when a low ability to control worries is observed (Caes et al., 2016). Associations between age and worry have also been shown in experimental research (Geronimi et al., 2016; Muris et al., 2002). Muris et al. (2002) demonstrated positive associations among age and cognitive development, worry elaboration, and the presence of a personal worry. Geronimi et al. (2016) report age-moderated relationships between working memory, planning, monitoring, and worry but only at younger ages. Whereas, this study showed a consistent degree of worrisome thoughts across the ages and a trend, albeit not significant, for the oldest children to more commonly report experiencing worry related to COVID-19. When considering the context of the present study, the pandemic management strategy in Sweden has had most direct impact on the oldest respondents given the decision to close gymnasiet (i.e., upper secondary school) sites and move education exclusively online.

### Methodological Considerations

There are benefits to using anonymous online surveys. For example, due to the anonymity, respondents are more inclined to discuss problems and provide honest feedback and there is little fear of embarrassment or reprisals, which can boost response rates. However, there are limitations associated with the method. A consequence of the researcher not having direct contact with the participant is a lack of control over how participation is conducted. There is a risk of respondents misreporting who they are, individuals retaking the survey, and not knowing whether or not others (e.g., parents) are assisting participants. An anonymous survey also precludes the opportunity to follow up with respondents to improve understanding, which is common in qualitative research to gather rich insights into the phenomenon being investigated. Related to this, survey-based qualitative responses tend to be shorter and lacking in detail compared with those generated through in-depth interviews or focus groups. Despite the presentation of the question about worrisome thoughts as a text response question, the phrasing of the question meant there was a risk of the children and adolescents providing closed responses (i.e., yes/no). Although many responded “no” to the question (4–12 year olds: 23%; 13–18 year olds: 20%), those who indicated the presence of worrisome thoughts all provided elaboration beyond “yes.” A further limitation is that respondents are denied the opportunity to ask for clarification of questions, which could lead to misinterpretation. One example for the present study is whether the child knew somebody with COVID-19. The children were given a “not sure” response option. Although the intention was for children to use this response option if they had not communicated with others about COVID-19 and therefore could be unaware of an acquaintance’s diagnosis, it could be the case that children used this response option because they did not understand the question. The online method also limits responses to those have an access to the Internet. Yet, parents, preschool personnel and school personnel were targeted as gatekeepers to provide access to the survey particularly for the younger children. It was important from an ethical stance point that caregivers had an awareness of the child’s participation in the survey, yet it could raise questions about potential bias in the children’s responses if the caregiver was able to see the responses.

The qualitative design gave children space to provide detail about their worries in their own words, which resulted in the somewhat unexpected findings regarding economic worries. Dependability of the qualitative analysis was established through co-rating the data at the code level, which resulted in an excellent level of interrater reliability. The data were securely stored in the Qualtrics system and the coding procedure well documented in excel files to enable someone outside the research to follow, audit, and critique the research process to ensure dependability and confirmability. The survey achieved good geographical representation with responses from all 21 counties in Sweden. For those counties that were overrepresented (Gotland and Uppsala), this was likely due to more extensive local media coverage. Sensitivity analyses showed that excluding the data from Uppsala and Gotland did not change the results of the statistical analyses. If you compare the distribution of housing among the children who participated in the study with population statistics, there is a smaller proportion who live in rental apartments: 26 % of children (0–12 years) and 23% of adolescents (13–19 years) in Sweden live in a rented apartment (Statistics Sweden, 2019) compared with 17% (4–12 years) and 16% (13–18 years) respectively in the study. Considering the known link between housing type and income in Sweden this might indicate that the children and adolescents in the study were coming from slightly better socioeconomic conditions than a representative sample of children and adolescents living in Sweden, which affects the generalizability of the findings. It could be that children in families with lower socioeconomic status have experienced differential worries. For example, COVID-19 food insecurities have been raised as a concern for lower socioeconomic groups (Ambrose, 2020). Yet, these inferences cannot be made within the scope of this study. A further representation issue that arose was the lack of responses on the alternative language versions of the survey. As the translations were available in PDF on the study webpage, as well as survey links, it could be the case that respondents used these as support aids to complete the Swedish survey. However, we cannot say for sure. It could therefore be the case that non-Swedish speaking groups were underrepresented in the survey.

### Implications of the Findings

The high proportion of children and adolescents reporting COVID-19-related worries supports the notion of universal mental health intervention. This aligns with current thinking that schools are a suitable environment for conducting mental health promotion (O’Connor et al., 2018). There was no indication of socioeconomic status or geographic area (urban vs. rural) affecting the presence of worrisome thoughts among children and adolescents in Sweden, which is supportive of the universal approach. Our findings indicate that interventions could be tailored for age. Adolescents reported worrisome thoughts about the global economy, as well as for their own employment prospects. There could be value in discussing financial aspects relating to the COVID-19 pandemic with this group. Financial literacy education research indicates that adolescents are naturally more interested in learning about financial issues they perceive as salient in their lives at that particular time (Amagir et al., 2018), which suggests they would be receptive to COVID-19 economy-based discussions. Whether these approaches would reduce COVID-19 worrisome thoughts and prevent the onset of mental ill health requires further research. Looking to existing evidence on mental health promotion in schools, longer-term, whole-school approaches appear to achieve greater impact (O’Reilly et al., 2018; Wells et al., 2003). Some examples of intervention formats include structured lesson plans, video-based learning, development of school personnel skills, and parent involvement (O’Reilly et al., 2018). Given the digital learning environment adopted by upper secondary schools in Sweden, and more broadly in the international context, some intervention formats (e.g., video-based learning) offer pragmatic advantages. Involving children and young people in the development of the intervention could be advantageous. In a qualitative evaluation conducted in Scotland, young people expressed a number of preferences for mental health intervention in schools including: use of structured talks, discussion, role‐play, and video; delivery by a familiar person; maintaining typical peer constellations; and preventive as well as reactive techniques (Woolfson et al., 2009).

This study adopted a qualitative methodology that intended to investigate worrisome thoughts among children and adolescents in the particular context of Sweden; hence generalizability of the findings to other international contexts was not an expected attribute. Other countries could take inspiration from these findings; however, evaluation of child and adolescent experiences in the local context is advised.

## Conclusions

Worry about “Corona” was common among children and adolescents in Sweden. Worrisome thoughts were mostly related to disease or death, not only connected to known COVID-19 risk groups but also to the children themselves. Some respondents also expressed existential worries and general worries for society. Universal preventative mental health intervention is warranted and could be conducted in the school setting. Intervention could be tailored by age, covering discussion on financial aspects with adolescents. Globalized media coverage could be connected to the worrisome thoughts and it would be interesting to learn more about the COVID-19 information sources utilized by children and adolescents in Sweden.
